# Influence of Paternalistic Leadership Style on Innovation Performance Based on the Research Perspective of the Mediating Effect of the Constructive Deviance of Employees

**DOI:** 10.3389/fpsyg.2021.719281

**Published:** 2021-10-13

**Authors:** Lin Li, Shiqian Wang

**Affiliations:** Faculty of Management, City College of Dongguan University of Technology, Dongguan, China

**Keywords:** paternalistic leadership, constructive deviance, innovation performance, mediating effect, high-tech enterprise

## Abstract

Innovation is the primary driving force behind the development of China as a modern economic power. This study examines the impact of paternalistic leadership on innovation, proposing a theoretical model using the three dimensions of paternalistic leadership (i.e., benevolence, morality, and authoritarianism) as independent variables, constructive deviance as a mediating variable, and innovation performance as the dependent variable. Empirical results showed that benevolent and moral leadership has a positive impact on innovation performance while authoritarian leadership has a negative impact. Constructive deviance by employees has a positive impact on innovation performance. Benevolent and moral leadership has a positive impact on the constructive deviance of employees, while authoritarian leadership has a negative impact on constructive deviance. In addition, benevolent and moral leadership has a positive impact on innovation performance through the constructive deviance of employees, while the impact of authoritarian leadership is negative. In practice, leaders should recognize that constructive deviance is a double-edged sword and guide employees to engage in reasonable constructive deviant behavior, thereby creating sound organizational environments to foster innovation, eliminate barriers, and benefit from the positive impact of the constructive deviance of employees to enhance innovation performance.

## Introduction

The modern development of China is driven by innovation. Some scholars hold that the individual innovation of employees is the source of enterprise innovation and is also the fundamental motivating force and foundation for improving enterprise innovation performance (Li et al., 2013). Knowledge workers are the most active core resources in the enterprise technological innovation system. Therefore, how to give full play to the creativity of knowledge workers and improve their innovation performance is becoming an increasingly critical concern. Leadership is also a core enterprise component and has an important influence on innovation and organizational performance (Ma and Zhang, 2018). Different management styles may have entirely different effects. With the growing emphasis on localization in management, paternalistic leadership has emerged as a management style characteristic of East Asian societies and contrasts with Western practices (Wu and Zhang, 2018). Paternalistic managers are influenced by traditional Chinese cultural concepts that reflect “mixed characteristics” involving authoritarian leadership, benevolent leadership, and moral leadership (Lin and Zhuang, 2014). Therefore, Western leadership theories cannot fully explain the leadership style and behavior of Chinese enterprises or their impact on innovation performance.

Employee deviance initially referred to destructive behavior that intentionally violates organization rules. However, the current view is more complex, with employee deviant behavior noticed as having both positive and negative impacts. Not all deviant behavior will damage the interests of an organization, and constructive deviant behavior may positively impact organizational innovation (Narayanan and Murphy, 2017). Constructive deviant behavior is an out-of-role behavior in which employees take the initiative to violate organizational norms to promote the interests of the organization (Kura et al., 2016). Although this kind of behavior deviates from organizational norms, it adheres to the moral standards of the organization and benefits the organization. By flexibly interpreting or following the organizational rules, employees can engage in innovative behavior (Mertens et al., 2016). Especially under more complex and unpredictable conditions, employees may follow the principles of the organization while still actively deviating from inappropriate organizational norms to promote the healthy development of the organization.

## Literature Review

### Social Exchange Theory

The social exchange theory was first proposed by Homans (1958). He pointed out that all human behaviors are exchange behaviors, such as material exchange and nonmaterial exchange, seeking to satisfy their needs and trying to achieve pay and return equivalence (Homans, 1958). Blau (1964) revised and developed the social exchange theory, pointing out that individuals engage in social interaction to maintain positive relationships, which are required to achieve reciprocity and promote the development of social exchange behavior. Blau divided exchange behavior into the economic exchange and social exchange, where the former is based on the exchange of interests, and the latter is based on the long-term mutually beneficial relationships and in-depth mutual trust. The social exchange theory provides an important theoretical basis for the proposed relationship among paternalistic leadership, the constructive deviance of employees, and innovation behavior. The constructive deviance of an employee will be influenced by interaction with others, and the close relationships between leaders and subordinates would result from employees feeling inspired by the benevolence and impartiality of leaders, forming a high-quality hierarchical exchange relationship. Employees are more willing to follow the leaders they find to be sympathetic, which will improve work engagement and promote exchange balance. This is beneficial to maintain the long-term sustainable mutually beneficial relationships between leaders and employees.

### Patriarchal Leadership Style and Innovation Performance

Farh and Cheng (2000) proposed the paternalistic leadership theory, noting that paternalistic leadership not only involves tolerance, love, justice, and integrity but also entails strict discipline and authority. They further added morality to the original two paternalistic leadership style dimensions of benevolence and authority. Farh and Cheng (2000) pointed out that subordinates feel grateful to benevolent leaders and respect moral leaders but feel obedience to and awe for authoritarian leadership. That is, different leadership styles lead to different reactions among subordinates, which is also the basis of analyzing the effectiveness of paternalistic leadership style.

Benevolent leaders are always solicitous to their subordinates, providing them with necessary resources and maintaining a close association with them (Huang et al., 2014). Benevolent leaders can not only increase the work enthusiasm of their subordinates but also stimulate them to provide positive opinions for decision-making (Lin and Zhuang, 2014). Moral leadership reflects excellent personal cultivation and integrity, and moral leaders set a good example for subordinates, make clear distinctions between public and private interests, and provide emphasized fairness and justice in decision-making, to maximize their credence and respect. Conversely, authoritarian leaders focus more on hierarchy, normally relying on institutional or personal authority to suppress subordinates, which will reduce the organizational identification and work enthusiasm of workers and will hinder innovation performance (Liu et al., 2017). Thus, this study proposes the following hypotheses:

H1: Benevolent leadership has a significant positive impact on innovation performance.

H2: Moral leadership has a significant positive impact on innovation performance.

H3: Authoritarian leadership has a negative impact on innovation performance.

### Constructive Deviance of Employees and Innovation Performance

The definition suggested by Galperin (2012) of constructive deviance holds that employees voluntarily violate organizational norms to promote the wellbeing of the organization. Constructive deviant behavior includes violating formal and nonformal regulations. Individual innovation performance refers to employees consciously creating, introducing, and applying new ideas and methods to achieve innovative work (Janssen and Van Yperen, 2004). Constructive deviant behavior means that employees need to break outdated organizational rules to improve organizational wellbeing. This type of behavior requires a creative mindset and can enhance individual innovation performance (Wang et al., 2018).

While workers may obey various rules and regulations to ensure overall normal operations, conforming to conventional practices and conventional ways of thinking may limit the implementation of pioneering ideas and innovative approaches. Constructive deviance means that employees need to break from outdated organizational rules and adopt new processes and norms for improving organizational value creation, thus enhancing motivation for organizational change and innovation (Kim and Choi, 2018). Constructive deviant behavior helps employees find benefits through exploring new possibilities. According to resource conservation theory, individuals seek to protect their access to resources. To maintain the exploratory advantage and obtain more resources, employees who implement constructive deviant behavior are often willing to attempt innovative ideas that can lead to improved innovation performance (Malik and Lenka, 2019). Wang et al. (2018) also pointed out that constructive deviant behavior can bring explorative advantages to implementers in some important fields. Constructive deviant behavior assists individuals in obtaining sufficient information and resources, develops divergent thinking, and adopts new ideas and practices, thus, further enhancing innovation performance. In addition, employees who carry out constructive deviant behaviors are regarded as change agents. These people actively share resources, establish trust relationships with other team members, and secure their supports for innovation (Cohen and Ehrlich, 2019). These supportive emotional resources from colleagues help to improve individual innovation performance.

H4: Constructive deviance of employees has a positive impact on innovation performance.

### Patriarchal Leadership Style and Constructive Deviance

While leaders have a certain degree of authority, their behaviors are subject to the expectations of organizational norms of employees and are easily observed and imitated by employees (Sheng and Yuxin, 2018). Thus, different leadership styles have different impacts on the innovative behavior of employees. Based on the social identity theory, this study discusses the impact of paternalistic leadership on deviant innovation behavior in Chinese organizations. Constructive deviant behavior refers to employees violating the organizational rules and regulations, seeking to improve the organizational wellbeing. The social identity theory examines harmonious relationships between employees and the organization, staff believe that their values, ideas, and practices within the organization are recognized as unique and positive, and these positive cognitions will encourage employees to engage in deviant innovation behavior.

Moral leadership refers to a set of behaviors that remain a good example for employees, clearly distinguish between public and private interests, and emphasize fairness and justice, resulting in the enthusiasm of employees being closely related to the moral conduct of their leaders (Cheng et al., 2004). Constructive deviant behavior entails employees violating organizational norms and regulations out of the belief that such behavior will ultimately benefit the organizational wellbeing. Based on the social identity theory, the self-perceptions, emotions, and behavior of employees are affected by organizational norms, examples, and views. An in-depth understanding of organizational goals and values will encourage the deviant innovation behavior of employees, by which they seek not only to benefit the organization but also to achieve personal goals.

To a certain extent, the centralization of authoritarian leadership results in decision-making based on personal judgment or preference instead of organizational system and rules, resulting in an unequal atmosphere within the organization (Zhang et al., 2018). The long-term centralization of authority and inequality will create an authoritarian environment (Ma and Zhang, 2018) in which the innovative ideas of employees are rejected by their leadership. This type of leadership actively discourages violations of organizational rules and regulations and thus deviant innovation behavior. Therefore, this study speculates that constructive deviant behavior is less likely to occur under authoritarian leadership:

H5: Benevolent leadership has a significant positive impact on constructive deviance.

H6: Moral leadership has a significant positive impact on constructive deviance.

H7: Authoritarian leadership has a significant negative impact on constructive deviance.

### The Mediating Role of Constructive Deviance

To ensure their authority, authoritarian leaders studiously maintain distance from employees and will avoid close exchanges with their subordinates, resulting in employees feeling a lack of psychological security or positive attitudes toward initiating innovation and team cooperation. The tolerance and support of benevolent leadership create a free atmosphere that supports subordinates, optimizing work results and efficiency, with leaders sharing with and learning from team members. Moral leaders rely on their charisma to influence and educate employees, encouraging them to fully identify with the organizational values and enhancing team cohesion and cooperation. Such leaders consider the psychological state of their employees, encouraging them to express their ideas and providing work resources and feedback, giving employees a strong sense of organizational attachment. The social exchange theory suggests that employees with a high organizational attachment will proactively maintain the interests of the organization, even if doing so requires actions that deviate from established norms (Li and Liu, 2014). Ming and Wenquan (2013) pointed out that leaders who treat employees favorably will arouse a strong sense of mission and responsibility toward the leaders and organization. This positive self-cognition may cause employees to engage in deviant behavior as a means of benefitting the organization. According to the active motivation model proposed by Parker et al. (2010), leadership style is an important work situational factor that can motivate employees to engage in active behavior beyond job responsibilities. Thus, this study proposes the following hypothesis:

H8: Constructive deviance plays a mediating role between patriarchal leadership and innovation performance.

### Research Method and Data Analysis

**Figure 1:**
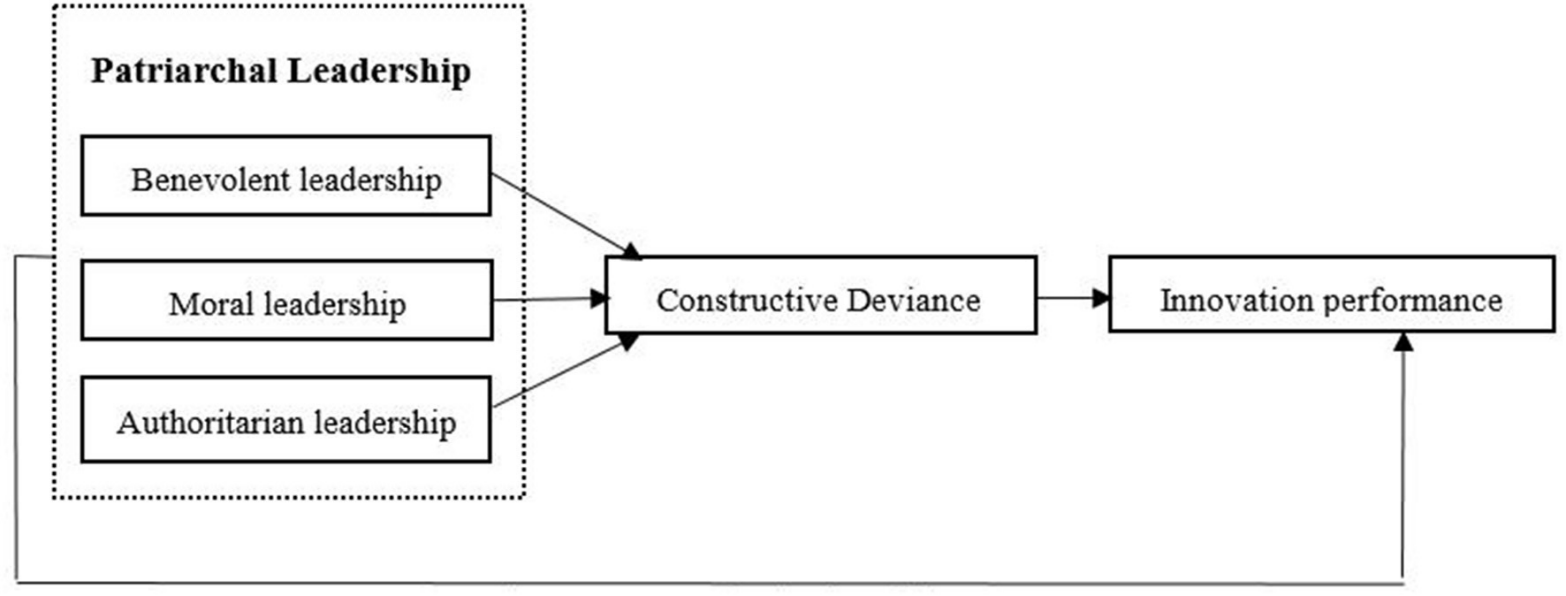
Hypothesized model.

Figure 1 illustrates the proposed conceptual framework.

### Variable Definitions

As shown in Table 1, existing variable definitions were adapted to match the research purpose.

**Table 1:** Definition.

**Name**	**Definition**	**Source**
Paternalistic leadership	Leadership behavior emphasizes strong discipline and authority, along with fatherly kindness and virtue.	Farh and Cheng (2000)
Benevolent leadership	Benevolent leadership focused on individualized and holistic concerns for the personal and familial wellbeing of subordinates.	Cheng et al. (2000)
Moral leadership	Leading by example and serving others in the public interest.	
Authoritarian leadership	Leaders who determine policy and make decisions autocratically, demanding the obedience and loyalty of subordinates.	
Constructive deviance	An out-of-role behavior by which employees violate organizational norms on their own initiative to promote organizational interests.	Kura et al. (2016)
Innovation performance	The proposing new and creative ideas of employees to improve organizational processes to enhance the significance, usefulness, and performance of the products and services of the organization.	Janssen (2000)

### Variable Measurement

Cheng et al. (2003) proposed a paternalistic leadership scale with three dimensions (i.e., benevolent leadership, moral leadership, and authoritarian leadership), with each dimension involving 5 items, for a total of 15 items.

Galperin (2012) proposed a constructive deviance scale with nine items, such as “In order to complete the task, sometimes I will deviate from the rules.”

Janssen and Van Yperen (2004) proposed an innovation performance scale including seven items, such as “Employees apply innovative ideas into practice” and “Employees always put forward some original solutions.”

### Methods and Procedures

The data were collected by questionnaire. SPSS 22 was used to perform the descriptive statistical analysis, and Pearson's correlation coefficient was used to investigate the relationships between variables (Statistical Product and Service Solutions, IBM SPSS Inc., Armonk, NY, USA^1^). The significance of the mediating effect was computed using PROCESS by Hayes (2012), using the bootstrapping technique. The sample included four high-tech and manufacturing companies in the Yangtze River Delta, with data gathered using online surveys, site surveys, and commissioned surveys. The online survey was conducted using the Questionnaire Star platform. The researcher established contact with the target enterprises, explaining the goals of the research, along with questionnaire instructions and anonymity assurances. The research process involved two steps, namely, pretest and formal test. The pretest was used to test the validity and comprehensibility of all questionnaire items. There were 60 pretest questionnaires distributed, of which 53 were received and analyzed. The pilot questionnaire was then modified to compose the final questionnaire, which was distributed in December 2020, with a total of 250 questionnaires distributed through various channels, of which 230 valid responses were received, for a response rate of 92%. Respondents were primarily aged under 35 years old and were 56.7% men. College graduates accounted for 63% of respondents, while 10% had a master's degree and 5% had PhDs. In terms of the employer, 47.2% of respondents worked at private companies, followed by 19.5% at state-owned enterprises and 17.7% at government/public institutions.

### Correlation Analysis

Table 2 summarizes the descriptive statistics and the results of correlation analysis.

**Table 2:** Correlation analysis.

	**1**	**2**	**3**	**4**	**5**
1. Benevolent leadership	1				
2. Moral leadership	0.209^***^	1			
3. Authoritarian leadership	−0.064	−0.048	1		
4. Constructive deviance	0.173^***^	0.157^***^	−0.389	1	
5. Innovation performance	0.338	0.109	−0.316^**^	0.148^**^	1
Mean	3.64	3.82	3.61	3.51	4.12
SD	0.244	0.197	0.228	0.257	0.285

* *P < 0.05, at 5% significance level*,

** *P < 0.01, at 1% significance level*,

*** *P < 0.001, at 0.1% significance level*.

### Reliability Test

The reliability of internal consistency was evaluated using Cronbach's alpha, with results as shown in Table 3, showing very good to excellent internal consistency for all items (Jietai et al., 2004).

**Table 3:** Reliability.

**Scale**	**Dimension**	**N**	**Cronbach's Alpha**
Paternalistic leadership	Benevolent leadership	5	0.883
	Moral leadership	5	0.711
	Authoritarian leadership	5	0.921
Constructive deviance		9	0.834
Innovation performance		7	0.805

### Hypotheses Testing

The results of correlation analysis indicate the preliminary verification of all hypotheses. To further verify the correlations, Mplus7.0 was used to analyze the hypothetical relationships. The five-factor model (i.e., benevolent leadership, moral leadership, authoritarian leadership, constructive deviance, and innovation performance) is found to have a high goodness of fit (2/df = 2.907 <3, root mean square error of approximation (RMSEA) = 0.057 <0.08, comparative fit index (CFI) = 0.941 > 0.9).

The results show that benevolent leadership has a significant positive relationship with innovation performance (β = 0.17, *p* < 0.05), moral leadership has a significant positive relationship with innovation performance (β = 0.23, *p* < 0.05), and authoritarian leadership has a significant negative relationship with innovation performance (β = −0.14, *p* < 0.05), thus confirming hypotheses H1, H2, and H3. Constructive deviance has a significant positive relationship with innovation performance (β = 0.27, *p* < 0.01), thus confirming hypothesis H4. Benevolent leadership has a significant positive relationship with constructive deviance (β = 0.48, *p* < 0.01), moral leadership has a significant positive relationship with constructive deviance (β = 0.35, *p* < 0.01), and authoritarian leadership has a significant negative relationship with constructive deviance (β = −0.30, *p* < 0.05), thus confirming hypotheses H5, H6, and H7.

The significance of the mediating effect was computed using PROCESS by Hayes (2012), using the bootstrapping technique. Table 4 shows the results for 5,000 sampling iterations and constructing 95% unbiased corrected CI.

**Table 4:** The result of mediating effect.

		**Estimate**	**SE**	**95% CI**	
				**Upper**	**Lower**
Indirect effect	Benevolent leadership–Constructive deviance–Innovation performance	0.053	0.025	0.121	0.016
	Moral leadership–Constructive deviance–Innovation performance	0.128	0.051	0.235	0.053
	Authoritarian leadership–Constructive deviance–Innovation performance	−0.085	0.035	−0.028	−0.138
Direct effect	Benevolent leadership–Innovation performance	0.333	0.082	0.537	0.324
	Moral leadership–Innovation performance	0.019	0.341	0.431	0.211
	Authoritarian leadership–Innovation performance	0.056	0.132	0.258	0.136
Total effect	Benevolent leadership–Innovation performance	0.386	0.106	0.277	0.143
	Moral leadership–Innovation performance	0.147	0.031	0.302	0.264
	Authoritarian leadership–Innovation performance	−0.029	0.025	0.239	0.002

Table 4 shows that, under the 95% CI, the bias correction CI of the indirect effect of benevolent leadership on innovation performance was [0.016, 0.121] excluding 0, which means that the mediating effect of constructive deviance between the two is significant and the effect is 0.053. In addition, the bias correction CI of the direct effect between benevolent leadership and innovation performance was [0.324, 0.537], also excluding 0, indicating that the direct effect is significant. Hence, constructive deviance has a partial mediating effect between benevolent leadership and innovation performance. The bias correction CI of the indirect effect of moral leadership on innovation performance was [0.053, 0.235] excluding 0, which means that the mediating effect of constructive deviance between the two is significant and the effect is 0.128. In addition, the bias correction CI of the direct effect between moral leadership and innovation performance was [0.211, 0.431], also excluding 0, indicating that the direct effect is significant. Hence, constructive deviance has a partial mediating effect between moral leadership and innovation performance. The bias correction CI of the indirect effect of authoritarian leadership on innovation performance was [−0.138, −0.028], excluding 0, which means that the mediating effect of constructive deviance between the two is significant and the effect is −0.085. In addition, the bias correction CI of the direct effect between authoritarian leadership and innovation performance was [0.136, 0.258], also excluding 0, indicating that the direct effect is significant. Hence, constructive deviance has a partial mediating effect between authoritarian leadership and innovation performance. Thus, hypothesis H8 is confirmed.

## Discussion and Conclusion

While paternalistic leadership with Chinese characteristics fits the present development situation in China, this study seeks to understand the relationship between paternalistic leadership and innovation performance. Prior research on factors that impact constructive deviance focuses mainly on work autonomy (Dietfried and Soren, 2015; Liu et al., 2017), organizational innovation climate (Wang et al., 2018), and employee creativity (Yang et al., 2019). Leadership style (Yi, 2018) should also be considered as an antecedent of constructive deviance. Few studies have examined the mediating effect of the constructive deviance of employees. This study explores the influence of constructive deviance on innovation performance, deepening our understanding of employee behavior and the quality of interaction between leaders and employees. Based on the social exchange theory, this study explored the impact of paternalistic leadership on innovation performance. Previous studies mainly focus on the self-awareness theory (Wang and Tian, 2019) and the self-determination theory (Wang and Tian, 2019) to explore mechanisms that impact employee innovation.

Based on the social exchange theory, this is an empirical study of the relationship between paternalistic leadership, constructive deviance, and innovation performance. The results provide scientific guidance and management suggestions for organizations to enhance competitiveness and build a harmonious organizational environment. Paternalistic leadership is found to have a positive impact on the innovation performance of employees, as does constructive deviance, which plays a mediating role between paternalistic leadership and innovation performance.

## Research Limitations and Future Directions

This study is subjected to certain limitations that potentially limit the generalizability of the findings. First, although the questionnaire was found to have high reliability and validity, it relies on subjective responses. Second, sampling is limited to a small number of high-tech enterprises in a specific industrial zone, and further studies should seek to verify the findings with different industries in different areas. Finally, this study considered only the constructive deviance of employees as a mediating variable between paternalistic leadership and innovation performance, but there may be other mediating variables with a significant effect that should be explored.

## Data Availability Statement

The raw data supporting the conclusions of this article will be made available by the authors, without undue reservation.

## Author Contributions

All authors were part of developing the measuring instrument. LL conceptualized this study, conducted the statistics, and wrote the manuscript. SW wrote the research methodology and constructive discussions. All authors contributed to the article and approved the submitted version.

## Funding

This work has been supported by City College of Dongguan University of Technology, China. The Research Program is a Study on the Mechanism of Employee Innovation Performance in the perspective of open innovation (No. 2020YZDYB05R).

## Conflict of Interest

The authors declare that the research was conducted in the absence of any commercial or financial relationships that could be construed as a potential conflict of interest.

## Publisher's Note

All claims expressed in this article are solely those of the authors and do not necessarily represent those of their affiliated organizations, or those of the publisher, the editors and the reviewers. Any product that may be evaluated in this article, or claim that may be made by its manufacturer, is not guaranteed or endorsed by the publisher.

## Footnotes

^1^IBM Corp. Released 2013. IBM SPSS Statistics for Windows, Version 22.0. Armonk, NY: IBM Corp.
